# The heart of physiological reports

**DOI:** 10.14814/phy2.15962

**Published:** 2024-03-05

**Authors:** Merry L. Lindsey, Keith R. Brunt, Petra Kleinbongard, Jason R. Carter, Crystal M. Ripplinger, Zamaneh Kassiri, Kara Hansell Keehan, Amanda J. LeBlanc, Jonathan A. Kirk

**Affiliations:** ^1^ School of Graduate Studies, Meharry Medical College Nashville Tennessee USA; ^2^ Research Service, Nashville VA Medical Center Nashville Tennessee USA; ^3^ Department of Pharmacology, Faculty of Medicine Dalhousie University Saint John New Brunswick Canada; ^4^ Institute for Pathophysiology, West German Heart and Vascular Center, University of Essen Medical School Essen Germany; ^5^ Robbins College of Health and Human Sciences Baylor University Waco Texas USA; ^6^ Department of Pharmacology, UC Davis School of Medicine Davis California USA; ^7^ Department of Physiology, Cardiovascular Research Center University of Alberta Edmonton Alberta Canada; ^8^ Associate Publications Director, Editorial and Content Development and Executive Editor, AJP‐Heart and Circulatory Physiology, American Physiological Society Rockville Maryland USA; ^9^ Department of Cardiovascular and Thoracic Surgery and Cardiovascular Innovation Institute, University of Louisville Louisville Kentucky USA; ^10^ Department of Cell and Molecular Physiology Loyola University Chicago Stritch School of Medicine Chicago Illinois USA

**Keywords:** cardiac, cardiovascular, editorial, physiology, vascular

It all started with the first issue in March of 2013 under the ground‐breaking and tireless leadership of the inaugural editor‐in‐chief Dr. Susan Wray, and since that time *Physiological Reports (Physiol Reports)* has published close to 4000 articles. The purpose of this editorial is to congratulate current editor‐in‐chief Dr. Josephine C. Adams and her team at *Physiol Reports* on their milestone 10‐year anniversary and to summarize the importance this journal has in the cardiovascular research community. As the executive editorial team of the *American Journal of Physiology: Heart and Circulatory Physiology (AJP Heart and Circ)*, it has been our pleasure to work closely with *Physiol Reports* in their cascade model to transfer manuscripts not accepted by our journal to our sister publication.

Established as a joint collaboration between the American Physiological Society and the Physiological Society, the mission of *Physiol Reports* is to publish sound science in basic, translational, and clinical physiology. With a 2022 Cite Score (Scopus) of 4 and a 2022 impact factor (Clarivate) of 2.5 and over 10,000 full text article views in 2023, *Physiol Reports* has garnered a robust reputation for solid publications. These numbers combined with a current overall acceptance rate of 77% and time to first decision at 20 days make this journal a strong option for publishing your cardiovascular research.

From inception, *AJP Heart and Circ* has been an active contributor to articles that we refer to *Physiol Reports*, and our intent is to continue providing this option. We often have submissions that have been rigorously reviewed and are meritorious in nature but do not quite reach the priority score needed to be competitive for publication in *AJP Heart and Circ*. In some cases, the findings of the article are deemed by reviewers as being too confirmatory, incremental, or observational without sufficient new mechanistic insight. The findings may be logical but not viewed as sufficiently impactful. Other times, researchers may be limited by funds, resources, or circumstances to perform additional experiments that reviewers have requested to further confirm the stated conclusions. The findings may be commendable for hypothesis generation or advance a specific area of study. For articles such as these, having the option to transfer to *Physiol Reports* is as reassuring to our editors as to our authors, as we want their efforts to result in publication and be received by the appropriate readership.

We have selected 10 recent articles published in *Physiol Reports* that are of high interest to the cardiovascular community (Table 1). These articles together illustrate the versatility and range within the cardiac and vascular topic areas that the journal publishes. If you have not read these articles or others published by *Physiol Reports*, we urge you to take a look.

**TABLE 1:** Ten recent publications in Physiological Reports that are of high interest to the cardiovascular research community.

Title	References
A new assessment method for right ventricular diastolic function using right heart catheterization by pressure–volume loop	Isotani et al. (2023)
Cardiomyocyte ZKSCAN3 regulates remodeling following pressure overload	Ouyang et al. (2023)
Evidence of sex differences in cancer‐related cardiac complications in mouse models of pancreatic and liver cancer	Gams et al. (2023)
Genetic background influences arterial vasomotor function in male and female mice	Holly et al. (2023)
Impact of bimonthly repeated total sleep deprivation and recovery sleep on cardiovascular indices	Pasetes et al. (2023)
Interleukin‐38 suppresses abdominal aortic aneurysm formation in mice by regulating macrophages in an IL1RL2‐p38 pathway‐dependent manner	Kurose et al. (2023)
Menopausal stage differences in endothelial resistance to ischemia–reperfusion injury	Delgado Spicuzza et al. (2023)
Mice with endothelial cell‐selective adhesion molecule deficiency develop coronary microvascular rarefaction and left ventricle diastolic dysfunction	Buncha et al. (2023)
Translational implications of bradyarrhythmia in hibernating brown bears	Gottlieb et al. (2023)
Visualization of cardiac uptake of bone marrow mesenchymal stem cell‐derived extracellular vesicles after intramyocardial or intravenous injection in murine myocardial infarction	Xu et al. (2023)

As Confucius said, “Wherever you go, go with all your heart.” Given the number of excellent articles on cardiovascular physiology published, *Physiol Reports* is doing just that. We congratulate *Physiol Reports* on an amazing journey as a trusted partner in establishing itself as a source of reliable physiology information and look forward to seeing what the next decade brings.

## CONFLICT OF INTEREST STATEMENT

The authors hold the position of the executive editorial board of *AJP Heart and Circ* and were blinded from reviewing or making decisions for this editorial. The authors have no other conflicts of interest to disclose.

## ETHICS STATEMENT

Not applicable.

## References

[phy215962-bib-0001] Buncha, V. , Fopiano, K. A. , Lang, L. , Williams, C. , Horuzsko, A. , Filosa, J. A. , Kapuku, G. , & Bagi, Z. (2023). Mice with endothelial cell‐selective adhesion molecule deficiency develop coronary microvascular rarefaction and left ventricle diastolic dysfunction. Physiological Reports, 11, e15643. 36946064 10.14814/phy2.15643 PMC10031300

[phy215962-bib-0002] Delgado Spicuzza, J. M. , Proctor, D. N. , Thijssen, D. H. J. , & Somani, Y. B. (2023). Menopausal stage differences in endothelial resistance to ischemia‐reperfusion injury. Physiological Reports, 11, e15768. 37734868 10.14814/phy2.15768 PMC10513907

[phy215962-bib-0003] Gams, A. , Nevarez, A. , Perkail, S. , Venegas, A. , George, S. A. , Efimova, T. , & Efimov, I. R. (2023). Evidence of sex differences in cancer‐related cardiac complications in mouse models of pancreatic and liver cancer. Physiological Reports, 11, e15672. 37102225 10.14814/phy2.15672 PMC10133859

[phy215962-bib-0004] Gottlieb, L. A. , Evans, A. L. , Fuchs, B. , Fröbert, O. , & Björkenheim, A. (2023). Translational implications of bradyarrhythmia in hibernating brown bears. Physiological Reports, 11, e15550. 36597216 10.14814/phy2.15550 PMC9810840

[phy215962-bib-0005] Holly, D. , Kim, H. , Woodman, C. R. , & Massett, M. P. (2023). Genetic background influences arterial vasomotor function in male and female mice. Physiological Reports, 11, e15824. 37771071 10.14814/phy2.15824 PMC10539628

[phy215962-bib-0006] Isotani, Y. , Amiya, E. , Hatano, M. , Kiriyama, H. , Uehara, M. , Ishida, J. , Tsuji, M. , Bujo, C. , Narita, K. , Ishii, S. , Kakuda, N. , Minatsuki, S. , Yagi, H. , Saito, A. , Numata, G. , Yamada, T. , Kurihara, T. , Suzuki, T. , & Komuro, I. (2023). A new assessment method for right ventricular diastolic function using right heart catheterization by pressure‐volume loop. Physiological Reports, 11, e15751. 37394657 10.14814/phy2.15751 PMC10315326

[phy215962-bib-0007] Kurose, S. , Matsubara, Y. , Yoshino, S. , Yoshiya, K. , Morisaki, K. , Furuyama, T. , Hoshino, T. , & Yoshizumi, T. (2023). Interleukin‐38 suppresses abdominal aortic aneurysm formation in mice by regulating macrophages in an IL1RL2‐p38 pathway‐dependent manner. Physiological Reports, 11, e15581. 36708509 10.14814/phy2.15581 PMC9884112

[phy215962-bib-0008] Ouyang, X. , Bakshi, S. , Benavides, G. A. , Sun, Z. , Hernandez‐Moreno, G. , Collins, H. E. , Kane, M. S. , Litovsky, S. , Young, M. E. , Chatham, J. C. , Darley‐Usmar, V. , Wende, A. R. , & Zhang, J. (2023). Cardiomyocyte ZKSCAN3 regulates remodeling following pressure‐overload. Physiological Reports, 11, e15686. 37144628 10.14814/phy2.15686 PMC10161215

[phy215962-bib-0009] Pasetes, L. N. , Rosendahl‐Garcia, K. M. , & Goel, N. (2023). Impact of bimonthly repeated total sleep deprivation and recovery sleep on cardiovascular indices. Physiological Reports, 11, e15841. 37849046 10.14814/phy2.15841 PMC10582224

[phy215962-bib-0010] Xu, C. M. , Sabe, S. A. , Brinck‐Teixeira, R. , Sabra, M. , Sellke, F. W. , & Abid, M. R. (2023). Visualization of cardiac uptake of bone marrow mesenchymal stem cell‐derived extracellular vesicles after intramyocardial or intravenous injection in murine myocardial infarction. Physiological Reports, 11, e15568. 36967241 10.14814/phy2.15568 PMC10040402

